# PP1γ promotes esophageal squamous cell carcinoma progression through the PP1γ/YAP1/SOX2 axis

**DOI:** 10.3389/fonc.2025.1586679

**Published:** 2025-06-23

**Authors:** Juan Du, Li Liu, Shutao Zheng, Chenglu Dai, Jingyu Liu, Wei Zhang, Hongwei Pu, Jing Xue

**Affiliations:** ^1^ State Key Laboratory of Pathogenesis, Prevention and Treatment of High Incidence Diseases in Central Asia, Department of Pathology, The First Affiliated Hospital of Xinjiang Medical University, Urumqi, China; ^2^ Department of Pathology, Changji People’s Hospital, Changji, Xinjiang, China; ^3^ State Key Laboratory of Pathogenesis, Prevention, Treatment of Central Asian High Incidence Diseases, Clinical Medical Research Institute, First Affiliated Hospital of Xinjiang Medical University, Xinjiang Uygur Autonomous Region, Urumqi, China; ^4^ School of Basic Medical Sciences, Xinjiang Medical University, Urumqi, China; ^5^ Department of Discipline Construction, The First Affiliated Hospital of Xinjiang Medical University, Urumqi, China; ^6^ Key Laboratory of Forensic Medicine, Xinjiang Medical University, Urumqi, China

**Keywords:** esophageal squamous cell carcinoma, PP1γ, immunohistochemistry, potential biological functions, prognosis

## Abstract

**Introduction:**

Esophageal squamous cell carcinoma (ESCC) is a highly aggressive malignancy with poor outcomes and limited targeted therapeutic options. While protein phosphatase 1γ (PP1γ) is overexpressed in various cancers, its role and mechanism in ESCC remains unclear. This study investigated the involvement of PP1γ in ESCC progression, particularly concerning YAP1 dephosphorylation and its regulation on stem cell markers.

**Methods:**

The expression levels of PP1γ, YAP1, SOX2, and NANOG in ESCC tissues and adjacent non-cancerous tissue samples were analyzed using bioinformatics and immunohistochemistry. Their association with clinical features and prognosis were also analyzed. Functional assays were performed in KYSE150 cells to assess the effects of PPP1CC silencing on cell proliferation, migration, and invasion. Western blotting and qRT-PCR were used to measure the expression of YAP1, phosphorylated YAP1 (p-YAP1), SOX2, and NANOG.

**Results and discussion:**

We found that PP1γ was highly expressed in ESCC and was significantly associated with poor prognosis, lymph node metastasis, and advanced pathological stages. Patients with high PP1γ levels had significantly shorter overall survival and progression-free survival (P < 0.05). In functional assays, silencing of PPP1CC in KYSE150 cells resulted in a marked decrease in cell proliferation, as measured by CCK-8 assays (P < 0.01). Colony formation assays confirmed the reduced colony-forming ability in PPP1CC-silenced cells (P < 0.01). Furthermore, Transwell invasion and migration assays demonstrated a significant reduction in both cell migration and invasion (P < 0.01). Western blot analysis revealed that silencing PPP1CC led to an increase in p-YAP1 and the ratio of p-YAP1 to YAP1, indicating inhibited YAP1 activity, alongside significant reductions in YAP1 and SOX2 protein levels (P < 0.05), while NANOG expression remained unchanged. This change was further confirmed by the qRT-PCR. Conclusively, PP1γ may promote ESCC progression by regulating YAP1 dephosphorylation and enhancing the expression of SOX2. The PP1γ/YAP1/SOX2 axis may provide potential therapeutic targets for ESCC treatment.

## 1 Introduction

Esophageal squamous cell carcinoma (ESCC) is one of the most aggressive malignancies of the digestive system, characterized by high rates of recurrence and metastasis (1). Despite advances in treatment strategies, the five-year survival rate remains dismal at only 15-25% (2). One of the major challenges in ESCC treatment is the lack of specific targeted therapies (3). Therefore, there is an urgent need to identify novel molecular targets for ESCC treatment.

The Hippo signaling pathway is a critical regulator of cell proliferation and tumorigenesis, particularly in squamous cell carcinomas (4). Yes-associated protein 1 (YAP1), a core effector of the Hippo pathway, is involved in tumor progression. After dephosphorylation, YAP1 is translocated to the nucleus where it activates downstream oncogenic transcription factors (5, 6). The role of YAP1 in various cancers is well-established (7, 8). One study has shown that YAP1 is overexpressed in ESCC tissues and plays a crucial role in regulating stemness (9). It is well known that dephosphorylated YAP1 translocates into the nucleus and induces the expression of target genes through interaction with homologous transcription factors. However, the mechanisms regulating the nuclear accumulation of YAP1 in ESCC remain unclear, particularly regarding the role of protein phosphatases.

Protein phosphatase 1 gamma (PP1γ) is a serine/threonine phosphatase involved in various cellular processes, including cell cycle regulation, DNA repair, and apoptosis (10, 11). It is highly expressed in many tumors and can promote tumor growth and progression (12, 13). However, the specific role of PP1γ in ESCC has not been fully elucidated. Emerging evidence suggests (13–16) that PP1γ may regulate YAP1 activity by regulating its dephosphorylation, thus contributing to tumor invasion and metastasis. Additionally, sex-determining region Y-box transcription factor 2 (SOX2) (17) and NANOG (18), which are markers of cancer stem cells, have been implicated in ESCC progression, but their regulation through PP1γ and YAP1 remains unclear.

This study aims to explore the role and mechanism of PP1γ in the progression of ESCC, focusing on its regulation of YAP1 dephosphorylation and its effect on stem cell makers like SOX2. Our findings may provide potential therapeutic targets for the treatment of ESCC.

## 2 Methods

### 2.1 Bioinformatics analysis using the Cancer Genome Atlas database

In the TCGA database, the expressions of PP1γ (gene name: PPP1CC), YAP1, SOX2, and NANOG in cancer tissues and adjacent non-cancerous tissues were analyzed using the ‘stats’ and ‘car’ packages in R software. Furthermore, the “survival,” “survminer,” “ggplot2,” and “proc” packages were used to analyze the prognosis and correlation of 168 cases of data from the TCGA. The proportional hazards assumption was assessed using the survival package, followed by Cox regression analysis. Gene Ontology (GO) and Kyoto Encyclopedia of Genomes (KEGG) pathway enrichment analysis were performed for differential genes using the “clusterProfiler” package.

### 2.2 Patient data and sample collection

Cancer tissues and their paired adjacent non-cancerous tissues were obtained from 107 patients with ESCC, who were treated in the Pathology Department of the First Affiliated Hospital of Xinjiang Medical University from November 2013 to June 2018. The clinical data of patients were collected, including gender, age, tumor size, differentiation degree, lymph node metastasis status, invasion depth, AJCC staging, presence of vascular invasion, and nerve invasion. This study was conducted in accordance with the Declaration of Helsinki and approved by the Ethics Committee of the First Affiliated Hospital of Xinjiang Medical University (approval no. K202412-56). Written informed consent was obtained from all patients.

### 2.3 Immunohistochemistry

Immunohistochemistry was conducted on 107 pairs of paraffin-embedded cancer tissues and their corresponding adjacent cancer tissues. Briefly, after deparaffinization, endogenous peroxidase inactivation, antigen retrieval, and blocking, the sections were incubated with primary antibodies of anti-PP1γ (ab245664; 1:200 dilution), anti-YAP1 (ab52771; 1:100 dilution), anti-SOX2 (ab93689; 1:100 dilution), and anti-NANOG (ab109250; 1:100 dilution) at 37°C for 1.5 hours. After washing, the incubation with the goat anti-rabbit IgG secondary antibody was conducted at 37°C for 1 hour. DAB staining was used for color development and hematoxylin was used for counterstaining.

The immunohistochemical staining results were evaluated using a double-blind method and scored based on the percentage of positive cells and the staining intensity. Under light microscopy, 5 random high-power fields (×400) of each slide were evaluated, with more than 300 cells counted in each field. The scoring criteria for the positive staining percentage were as follows: 0 points (<1%), 1 point (1-10%), 2 points (10-50%), 3 points (50-75%), and 4 points (>75%). Staining intensity was also assessed, with 0 points for negative staining, 1 point for weakly positive, 2 points for moderately positive, and 3 points for strongly positive. A combined total score was calculated: scores of 0–3 indicated low expression, while scores of 4–7 indicated high expression (19).

### 2.4 Cell lines

The human ESCC cell lines KYSE30, KYSE150, and KYSE450 were purchased from the Cell Bank of the Chinese Academy of Sciences (Shanghai, China). They were cultured in the DMEM (high glucose) Medium (C11995500BT, GIBCO, USA) containing10% fetal bovine serum and 1% streptomycin (15070-063, GIBCO, USA) at 37°C with 5% CO2.

### 2.5 Lentiviral-mediated transfection of shRNA-PPP1CC

PPP1CC expression was silenced in KYSE150 using lentiviral-mediated transfection of shRNA-PPP1CC. Briefly, the design and packaging of the lentiviral plasmids were performed by Shanghai Genechem Co., Ltd (Shanghai, China). These plasmids were constructed by cloning shRNA-PPP1CC-131619-1 (shRNA: CAAATGCCACGAGACCTGTAA), shRNA-PPP1CC-131620-1 (shRNA: ACATTTGGTGCAGAAGTGGTT), shRNA-PPP1CC-131621-1 (shRNA: GCGAATTATGCGACCAACTGA), and the negative control shRNA (shRNA-NC) into the GV493 vector (component sequence: hU6-MCS-CBh-gcGFP-IRES-puromycin). The KYSE150 cell was transfected with these lentiviral plasmids at an MOI of 20. A control group without lentiviral-mediated transfection was set up. At 48 hours and 72 hours after transfection, the fluorescence of GFP, a reporter for assessing transfection efficiency (20, 21), was observed under inverted fluorescence microscopy with 488 nm excitation light. The silencing efficiency of shRNA constructs was validated by Western blot.

### 2.6 Cell Counting Kit-8 assay

KYS150 cells were seeded into a 96-well plate and transfected with lentiviral plasmids as above described. After transfection for 72 hours, cells were incubated with 10% CCK-8 for 1 hour. Finally, the optical density at 450 nm (OD450) was measured using a microplate reader.

### 2.7 Colony formation assay

The KYS150 cells were transfected as above described. After transfection for 2 weeks, cells were seeded into a 6-well plate at a density of 1000 cells per well. After culturing for 14 days and when colonies were visible, the cells were fixed with 1 mL of methanol for 15 minutes. Subsequently, 0.1% crystal violet staining solution was added and incubated for 10–30 minutes. Finally, the cells were observed under a microscope. The number of colonies in each group was counted.

### 2.8 Transwell invasion and migration assay

KYS150 cells from each group were re-suspended in a serum-free medium and seeded in the upper chamber of the Transwell insert (Corning, USA) at a density of 5×10^5^ cells per well. This cell count was determined through preliminary experiments. The complete culture medium containing fetal bovine serum was added to the lower chamber. For cell invasion assay, the upper chamber of the Transwell insert was pre-coated with Matrigel (Beyotime Biotechnology Co., LTD, Shanghai, China). After culturing for 48 hours, the non-migrated/invaded cells on the upper surface of the upper chamber were wiped off and those on the lower surface were fixed with 4% formaldehyde at room temperature for 30 minutes. Then, the cells were stained with 0.1% crystal violet for 5 minutes, observed, and counted under a microscope.

### 2.9 Western blot analysis

Total proteins were extracted from KYSE150 cells of each group after lysis with RIPA (Boster, Wuhan, China). The protein concentration was determined using the BCA protein assay kit (Boster, China). After electrophoresis, the proteins were transferred onto a PVDF membrane. The membrane was blocked with 5% non-fat milk for 2 hours and then incubated with primary antibodies of anti-PP1γ (ab245664, Abcam, 1:5000), anti-phosphorylated YAP1 (p-YAP1) (ab76252, Abcam, 1:20000), anti-SOX2 (ab93689, Abcam, 1:1000), anti-NANOG (ab109250, Abcam, 1:5000), anti-YAP1 (ab52771, Abcam, 1:1000), and anti-GAPDH (ab9485, Abcam, 1:2000) at 4°C overnight. After washing, the incubation with Goat Anti-Rabbit IgG H&L (HRP) secondary antibodies (ab205718, Abcam, USA) was conducted for 2 hours at room temperature. Color development was performed using the ECL chemiluminescence method. GAPDH served as a loading control. The grayscale values of each protein were determined using Image J software.

### 2.10 qRT-PCR

The Trizol method was applied to extract RNA from the cells, and the extracted RNAs were reverse transcribed into cDNA. The qRT-PCR was performed using the 5X All-In-One RT MasterMix (with AccuRT Genomic DNA Removal Kit) (G492, ABM, Canada), according to the kit instructions. GAPDH was used as an endogenous control. The relative expression level of the target gene was calculated using the 2^-ΔΔCT^ method. The primer sequences were as follows: YAP1-F: TAGCCCTGCGTAGCCAGTTA, YAP1-R: TCATGCTTAGTCCACTGTCTG; Sox2-F: GCCGAGTGGAAACTTTTGTCG, Sox2-R: GGCAGCGTGTACTTATCCTTCT; and, GAPDH-F TGTTGCCATCAATGACCCCTT, GAPDH-R CTCCACGACGTACTCAGCG.

### 2.11 Statistical analysis

SPSS 25.0 was used for statistical analysis. The correlation between protein expression was analyzed by Spearman correlation. Kaplan-Meier curve method and Log-rank test were used for survival analysis. The median survival time and its 95% confidence interval (CI) were recorded. Univariate Cox regression analysis was applied for initial variable screening. Variables P < 0.1 were included in the subsequent multivariable Cox regression analysis. A backward stepwise selection method with a retention threshold of P < 0.05 was used to identify independent prognostic factors. Measurement data are expressed as the mean ± standard deviation and the comparison among multiple groups was analyzed by one-way analysis of variance. Categorical data are presented as numbers and percentages, and were analyzed by chi-square test. The differences were considered to be significant with a p-value less than 0.05.

## 3 Results

### 3.1 Bioinformatics analysis of PP1γ, YAP1, SOX2, and NANOG gene expressions in ESCC and the association of PP1γ with clinical features

**Figure 1:**
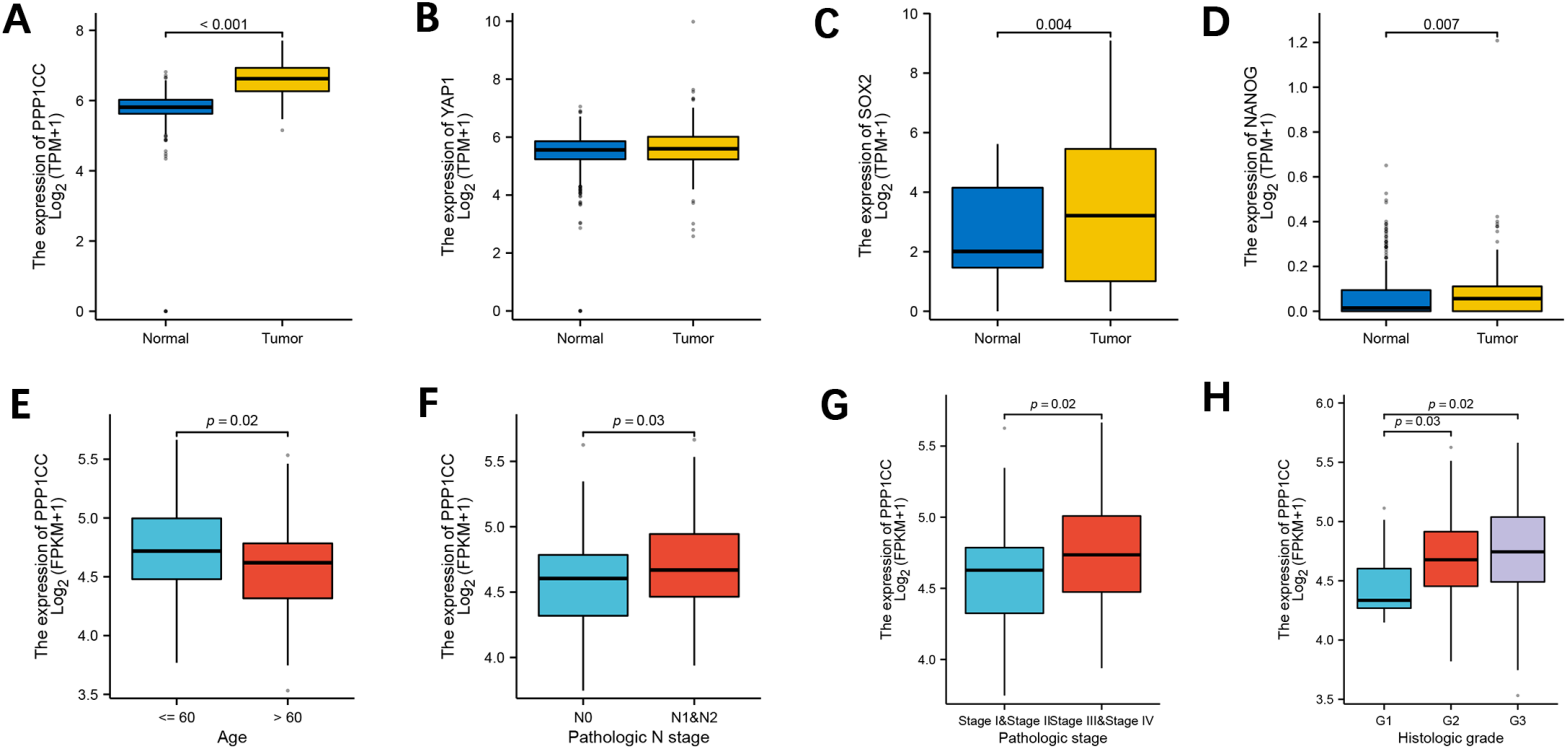
Expression of PPP1CC, YAP1, SOX2, and NANOG and the association of PPP1CC with clinical characteristics. The expressions of PPP1CC, YAP1, SOX2, and NANOG in ESCC tumor and normal tissues from the TCGA database were analyzed using bioinformatics. **(A-D)** Compared with normal tissues, PPP1CC **(A)**, YAP1 **(B)**, SOX2 **(C)**, and NANOG **(D)** were highly expressed in ESCC tissues. **(E-H)** The association between PPP1CC expression and clinical pathological indicators of ESCC, including **(E)** age; **(F)** lymph node metastasis; **(G)** pathological staging; and **(H)** histological grading.

We analyzed the gene expressions of PP1γ, YAP1, SOX2, and NANOG in ESCC and adjacent non-cancerous tissues (normal controls) through bioinformatics analysis of the TCGA database. The results revealed that the expressions of PP1γ (**Figure 1A**), SOX2 (**Figure 1C**), and NANOG (**Figure 1D**) were significantly elevated in ESCC tissues compared to normal controls (P < 0.05). YAP1 expression was also higher in ESCC, however, the difference was not statistically significant (P > 0.05) (**Figure 1B**). Additionally, PP1γ was significantly highly expressed in patients with age ≤ 60 years (**Figure 1E**), lymph node metastasis N1&N2 (**Figure 1F**), pathological stage III&IV (**Figure 1G**), and histological Grade 2&3 (**Figure 1H**) (P <0.05), indicating its association with age, lymph node metastasis, pathological staging, and histological grading.

### 3.2 High PP1γ expression is associated with poor survival of ESCC patients from the TCGA database

**Figure 2:**
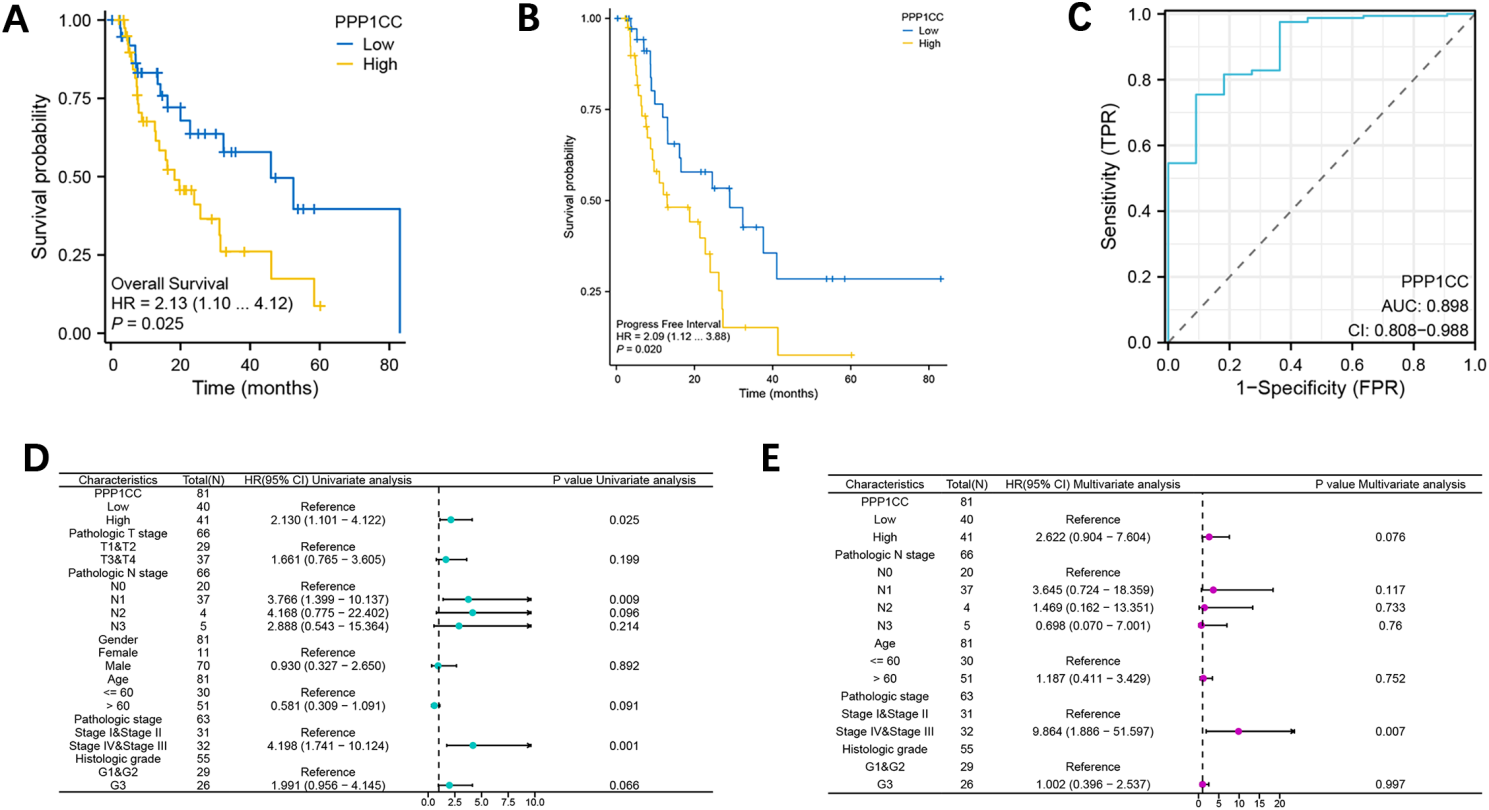
The expression of PPP1CC is associated with the prognosis of ESCC patients. Patient survival was analyzed with the Kaplan-Meier method. **(A, B)** The Kaplan-Meier survival curves of 5-year OS **(A)** and 5-year PFS in patients with high and low PPP1CC. **(C)** The ROC curve of PPP1CC in predicting the 1-year OS of ESCC patients. The univariate **(D)** and multivariate **(E)** Cox regression analyses to identify prognostic factors for ESCC.

Kaplan-Meier curve and Log-rank test further demonstrated that patients with high PP1γ expression had shorter 5-year overall survival (OS) (median: 18.6 months; 95% CI: 1.10–4.12) (**Figure 2A**) and 5-year progression-free survival (PFS) (median: 13.2 months; 95% CI: 1.12–3.88) (**Figure 2B**) (P < 0.05). ROC curve analysis revealed that PP1γ displayed good performance in predicting the 1-year OS of patients, with an AUC of 0.898 (**Figure 2C**). Univariate and multivariate Cox regression analyses identified PP1γ expression and advanced clinical stage as independent prognostic factors for ESCC (P < 0.05) (**Figures 2D, E**).

### 3.3 Correlation analysis of PP1γ, YAP1, SOX2, and NANOG expression in ESCC

**Figure 3:**
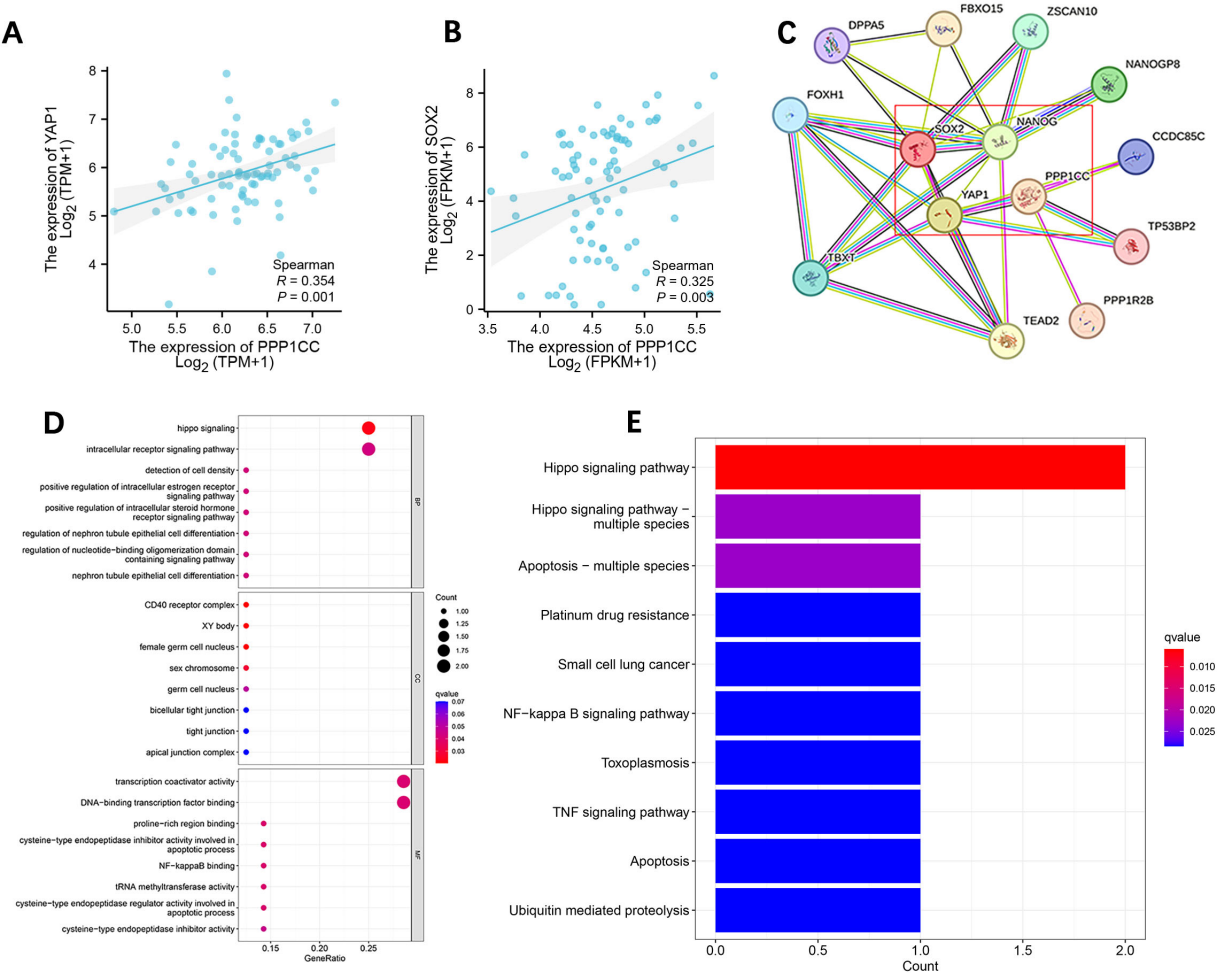
Correlation analysis of PPP1CC, YAP1, SOX2, and NANOG in ESCC and GO and KEGG enrichment analysis. **(A, B)** PPP1CC was positively correlated with the expression of YAP1 **(A)** and SOX2 **(B, C)** Protein-protein interaction network showing the relationship between PPP1CC, YAP1, SOX2, and NANOG. **(D, E)** GO annotation **(D)** and KEGG pathway enrichment analysis **(E)**.

The Spearman correlation analysis by R software showed that the expression of PP1γ (r=0.325, P=0.003) was positively correlated with YAP1 and SOX2 (r=0.325, P=0.003) (**Figures 3A, B**). Thirteen genes co-expressed with PP1γ (gene name: PPP1CC) were identified using the STRING database, among which YAP1, SOX2, and NANOG had an interaction relationship with PP1γ (**Figure 3C**). GO enrichment analysis of these genes showed that YAP1 was mainly involved in the regulation of signaling pathways and cell differentiation (**Figure 3E**). The enriched GO items in cell components included sex chromosomes and female reproductive nuclei, and those in molecular functions included transcriptional coactivator activity, tRNA methyltransferase activity, and cysteine-type endopeptase regulator activity involved in cell apoptosis. Moreover, KEGG pathway enrichment analysis showed that YAP1 was mainly involved in the Hippo signaling pathway, TNF signaling pathway, and NF-kappa B signaling pathway (**Figure 3D**).

### 3.4 Immunohistochemical analysis of PP1γ, YAP1, SOX2, and NANOG in ESCC tissues and their association with patient clinical features and survival

**Figure 4:**
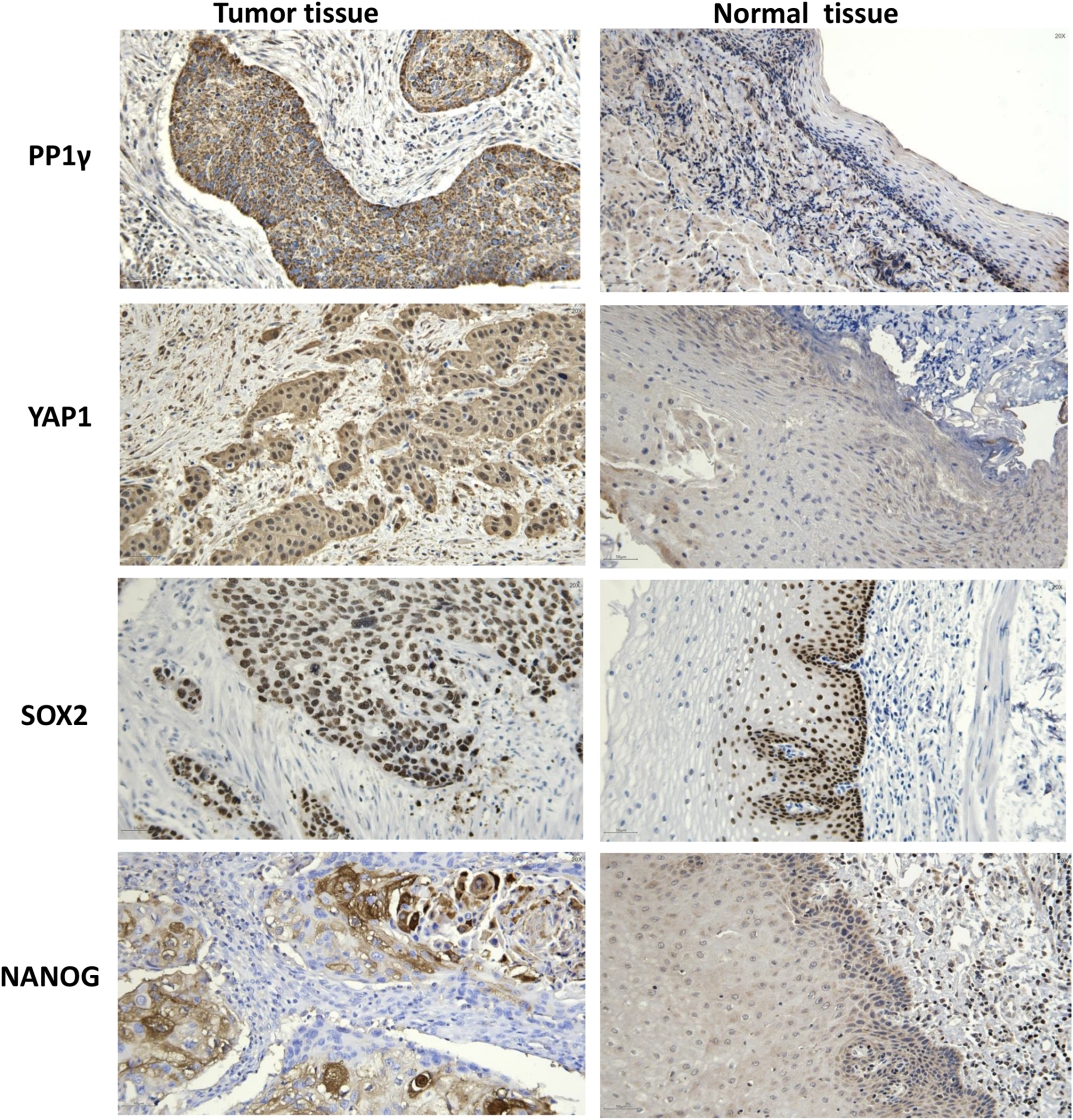
Immunochemistry analysis of PP1γ, YAP1, SOX2, and NANOG proteins in ESCC tissues and adjacent non-cancerous tissues. Immunochemistry was conducted to detect the expression of PP1γ, YAP1, SOX2, and NANOG in ESCC tissues and adjacent non-cancerous tissues. Representative immunohistochemistry results are shown.

Immunohistochemical staining of tissue samples confirmed high expression levels of PP1γ, YAP1, SOX2, and NANOG proteins in tumor tissues compared to adjacent non-cancerous tissues (**Figure 4**) (**Table 1**).

**Table 1:** Expression levels of PP1γ, YAP1, SOX2, and NANOG proteins in tissues.

Samples	Number	PP1γ		YAP1		SOX2		NANOG	
		Low expression	High expression	Low expression	High expression	Low expression	High expression	Low expression	High expression
Tumor tissue	107	38(35.5)	69(64.5)	30(28.0)	77(71.9)	40(37.3)	67(62.6)	45(42.1)	62(57.9)
Normal tissue	107	97(90.6)	10(9.3)	80(74.7)	27(25.2)	70(65.4)	37(34.5)	60(56.1)	47(43.9)
P		0.01		0.01		0.023		0.01	

Data are expressed as n (%). The chi-square test was used for statistical analysis.

Moreover, to determine the association of these proteins with clinical pathological features, a chi-square test was performed. The results indicated that high PP1γ expression was significantly associated with lymph node metastasis (P = 0.004), while high YAP1 expression was significantly related to male gender (P = 0.042), lymph node metastasis (P < 0.01), and advanced AJCC stage (P = 0.012) (**Table 1**). Similarly, SOX2 and NANOG expression were both significantly associated with lymph node metastasis (P < 0.01) and advanced AJCC stage (P = 0.016 and P = 0.031, respectively) (**Table 2**).

**Table 2:** The relationship between the expression of PP1γ, SOX2, YAP1, and NANOG and clinical pathological features.

Clinical pathological features	Number	PP1γ			YAP1			SOX2			NANOG		
		Low expression	High expression	*P*	Low expression	High expression	*P*	Low expression	High expression	*P*	Low expression	High expression	*P*
Age				0.85			0.357			0.473			0.464
≤65	55	20 (36.3)	35 (63.6)		26 (47.2)	29 (52.7)		26 (47.2)	29 (52.7)		25 (45.4)	30 (54.5)	
>65	52	18 (34.6)	34 (65.3)		20 (38.4)	32 (61.5)		21 (40.3)	31 (59.6)		20 (38.4)	32 (61.5)	
Gender				0.902			0.042			0.139			0.081
Female	33	12 (36.3)	21 (63.6)		19 (57.5)	14 (42.4)		18 (54.5)	15 (45.4)		18 (54.5)	15 (45.4)	
Male	74	26 (35.1)	48 (64.8)		27 (36.4)	47 (63.5)			45 (60.8)		27 (36.4)	47 (63.5)	
Tumor size				0.136			0.133			0.353			0.25
≤3cm	25	12 (48.0)	13 (52.0)		14 (56.0)	11 (44.0)		13 (52.0)	12 (48.0)		13 (52.0)	12 (48.0)	
>3cm	82	26 (31.7)	56 (68.2)		32 (39.0)	50 (60.9)		34 (41.4)	48 (58.5)		32 (39.0)	50 (60.9)	
Differentiation degree				0.659			0.458			0.449			0.461
Low	16	7 (43.7)	9 (56.2)		5 (31.2)	11 (68.7)		5 (31.2)	11 (68.7)		5 (31.2)	11 (68.7)	
Middle	76	25 (32.8)	51 (67.1)		33 (43.4)	43 (56.5)		34 (44.7)	42 (55.2)		32 (42.1)	44 (57.8)	
High	15	6 (40.0)	9 (60.0)		8 (53.3)	7 (46.6)		8 (53.3)	7 (46.6)		8 (53.3)	7 (46.6)	
Depth of invasion				0.297			0.639			0.497			0.563
Mucous layer	3	2 (66.6)	1 (33.3)		2 (66.6)	1 (33.3)		1 (33.3)	2 (66.6)		1 (33.3)	2 (66.6)	
Muscle layer	39	11 (28.2)	28 (71.7)		17 (43.5)	22 (56.4)		20 (51.2)	19 (48.7)		19 (48.7)	20 (51.2)	
Full-thickness	65	25 (38.4)	40 (61.5)		26 (40.0)	39 (60.0)		26 (40.0)	39 (60.0)		25 (38.4)	40 (61.5)	
Lymphatic metastasis				0.004			<0.01			<0.01			<0.01
No	65	30 (46.1)	35 (53.8)		39 (60.0)	26 (40.0)		39 (60.0)	26 (40.0)		37 (56.9)	28 (43.0)	
Yes	42	8 (19.0)	34 (80.9)		7 (16.6)	35 (83.3)		8 (19.0)	34 (80.9)		8 (19.0)	34 (80.9)	
AJCC stage				0.833			0.012			0.016			0.031
IB	25	10 (40.0)	15 (60.0)		17 (68.0)	8 (32.0)		17 (68.0)	8 (32.0)		16 (64.0)	9 (36.0)	
IIA, B	77	26 (33.7)	51 (66.2)		28 (36.3)	49 (63.6)		29 (37.6)	48 (62.3)		28 (36.3)	49 (63.6)	
IIIA, B, C	5	2 (40.0)	3 (60.0)		1 (20.0)	4 (80.0)		1 (20.0)	4 (80.0)		1 (20.0)	4 (80.0)	
Vascular infiltration				0.894			0.268			0.232			0.125
No	88	31 (35.2)	57 (64.7)		40 (45.4)	48 (54.5)		41 (46.5)	47 (53.4)		40 (45.4)	48 (54.5)	
Yes	19	7 (36.8)	12 (63.1)		6 (31.5)	13 (68.4)		6 (31.5)	13 (68.4)		5 (26.3)	14 (73.6)	
Nerve invasion				0.274			0,481			0.871			0.717
No	85	28 (32.9)	57 (67.0)		38 (44.7)	47 (55.2)		37 (43.5)	48 (56.4)		35 (41.4)	50 (58.8)	
Yes	22	10 (45.4)	12 (54.5)		8 (36.3)	14 (63.6)		10 (45.4)	12 (54.5)		10 (45.4)	12 (54.5)	

Data are expressed as n (%). The chi-square test was used for statistical analysis.

Spearman correlation analysis revealed that PP1γ expression was positively correlated with YAP1 (r = 0.460, P < 0.01), SOX2 (r = 0.327, P = 0.001), and NANOG (r = 0.317, P = 0.001) (**Table 3**).

**Table 3:** Correlation analysis of PP1γ expression with YAP1, SOX2, and NANOG in ESCC.

Protein	Correlation analysis	PP1γ		r	P
	Expression level	Low expression	High expression		
YAP1	Low expression	28 (26.1)	18 (16.8)	0.460	<0.01
	High expression	10 (9.3)	51 (47.6)		
SOX2	Low expression	25 (23.3)	22 (20.5)	0.327	0.001
	High expression	13 (12.1)	47 (43.9)		
NANOG	Low expression	24 (22.4)	21 (19.6)	0.317	0.001
	High expression	14 (13.1)	48 (44.8)		

Spearman correlation was used for correlation analysis.

**Figure 5:**
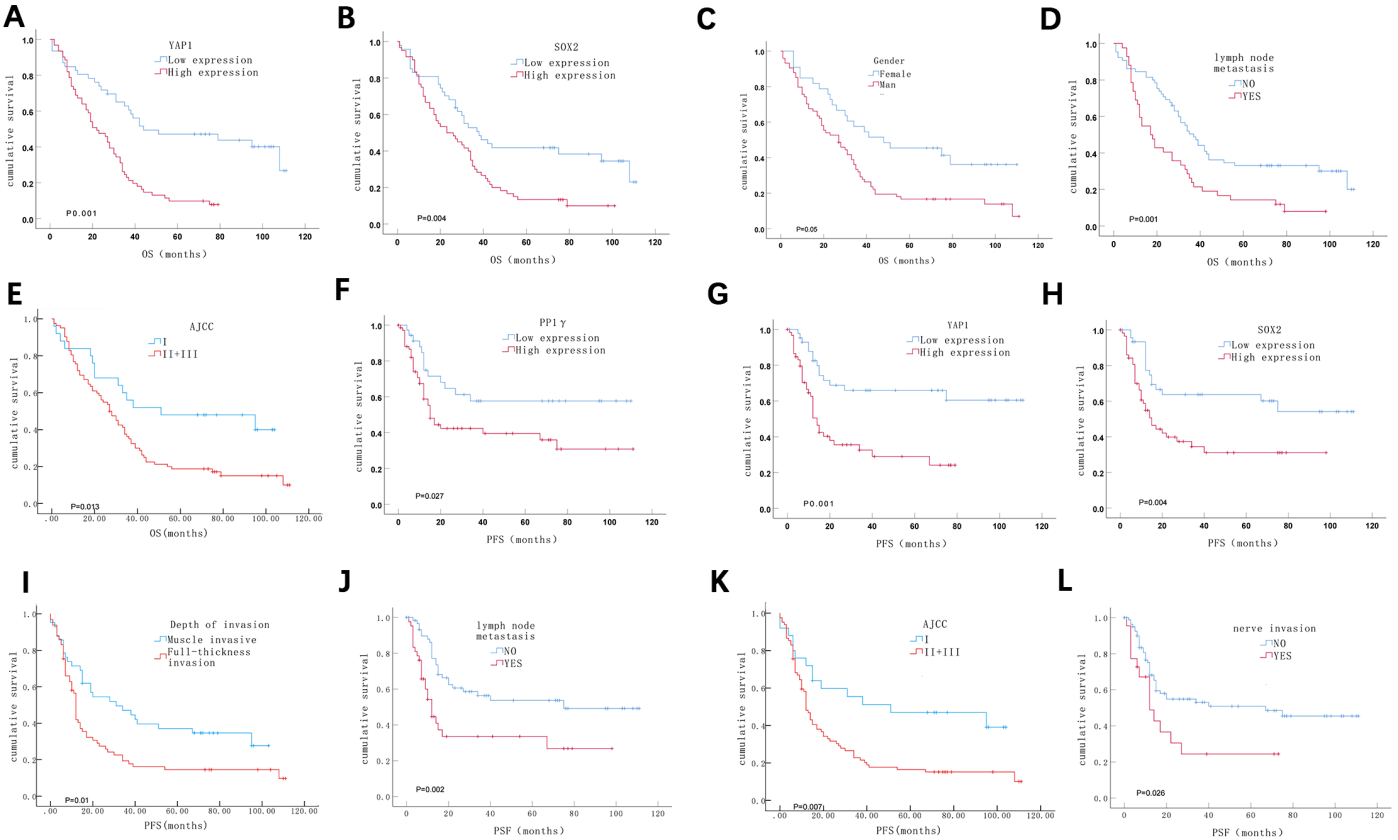
Kaplan-Meier survival analysis of ESCC patients. **(A-E)** ESCC patients with high expression of YAP1 **(A)** and SOX2 **(B)**, male gender **(C)**, lymph node metastasis **(D)**, and a later pathological stage **(E)** had a shorter OS. **(F-L)** ESCC patients with high expression of PP1γ **(F)**, YAP1 **(G)**, and SOX2 **(H)**; deeper depth of invasion **(I)**; lymph node metastasis **(J)**; a later pathological stage **(K)**; and neural invasion **(L)** experienced shorter PFS.

Additionally, survival analysis showed that patients with high expression of YAP1 and SOX2, male gender, lymph node metastasis, and advanced AJCC stage had significantly shorter OS (P < 0.05). Specifically, for patients with high levels of YAP1 expression, the median OS was 23.0 months (95% CI:15.0–30.9), compared to 42.0 months (95% CI: 19.8–88.5) for those with low expression (**Figure 5A**). Similarly, patients with high SOX2 expression had a median OS of 28.0 months (95% CI: 17.6–38.4), while those with low SOX2 expression had a median OS of 45.0 months (95% CI: 7.4–98.1) (**Figure 5B**). In terms of gender, male patients experienced a median OS of 27.0 months (95% CI: 16.6–37.4), compared to 48.0 months (95% CI: 5.0–91.0) for females (**Figure 5C**). Those with lymph node metastasis had a median OS of 17.0 months (95% CI: 10.6–23.3), versus 37.0 months (95% CI: 30.3–43.7) for patients without metastasis (**Figure 5D**). Patients in the advanced AJCC stage had a median OS of 27.0 months (95% CI: 20.0–33.8), whereas those in earlier stages had a median OS of 51.0 months (95% CI: 13.4–124.4) (**Figure 5E**). Meanwhile, patients with high expression of PP1γ, YAP1, and SOX2, tumor invasion depth, lymph node metastasis, advanced AJCC stage, and nerve invasion had significantly shorter PFS (P < 0.05). In detail, patients exhibiting high PP1γ expression had a median PFS of 15.0 months (95% CI: 11.4–18.2) (**Figure 5F**). The median PFS for high YAP1 expression was 14.0 months (95% CI: 11.3–16.6) (**Figure 5G**) while for high SOX2 expression, it was 15.0 months (95% CI: 9.3–20.6) (**Figure 5H**). Notably, the median PFS and its 95% CI could not be obtained for patients exhibiting low expression of PP1γ, YAP1, and SOX2, due to a recurrence rate of less than 50%. The median PFS for patients with greater tumor invasion depth was 12.0 months (95% CI: 11.1–17.2), compared to 31.0 months (95% CI: 7.6–54.3) for those with lesser invasion (**Figure 5I**). Likewise, for lymph node metastasis, the median PFS was 12.0 months (95% CI: 8.7–15.2) versus 75.0 months (95% CI: 19.7–128.8) for patients without metastasis (**Figure 5J**). Patients with advanced AJCC stages experienced a median PFS of 12.0 months (95% CI: 9.8–14.1), compared to 51.0 months (95% CI: 6.5–78.4) for earlier stages (**Figure 5K**). Finally, those with nerve invasion had a median PFS of 18.0 months (95% CI: 7.8–42.3) versus 47.0 months (95% CI: 11.3–86.4) in those without nerve invasion (**Figure 5L**).

### 3.5 PPP1CC is successfully silenced in KYSE150 cells

**Figure 6:**
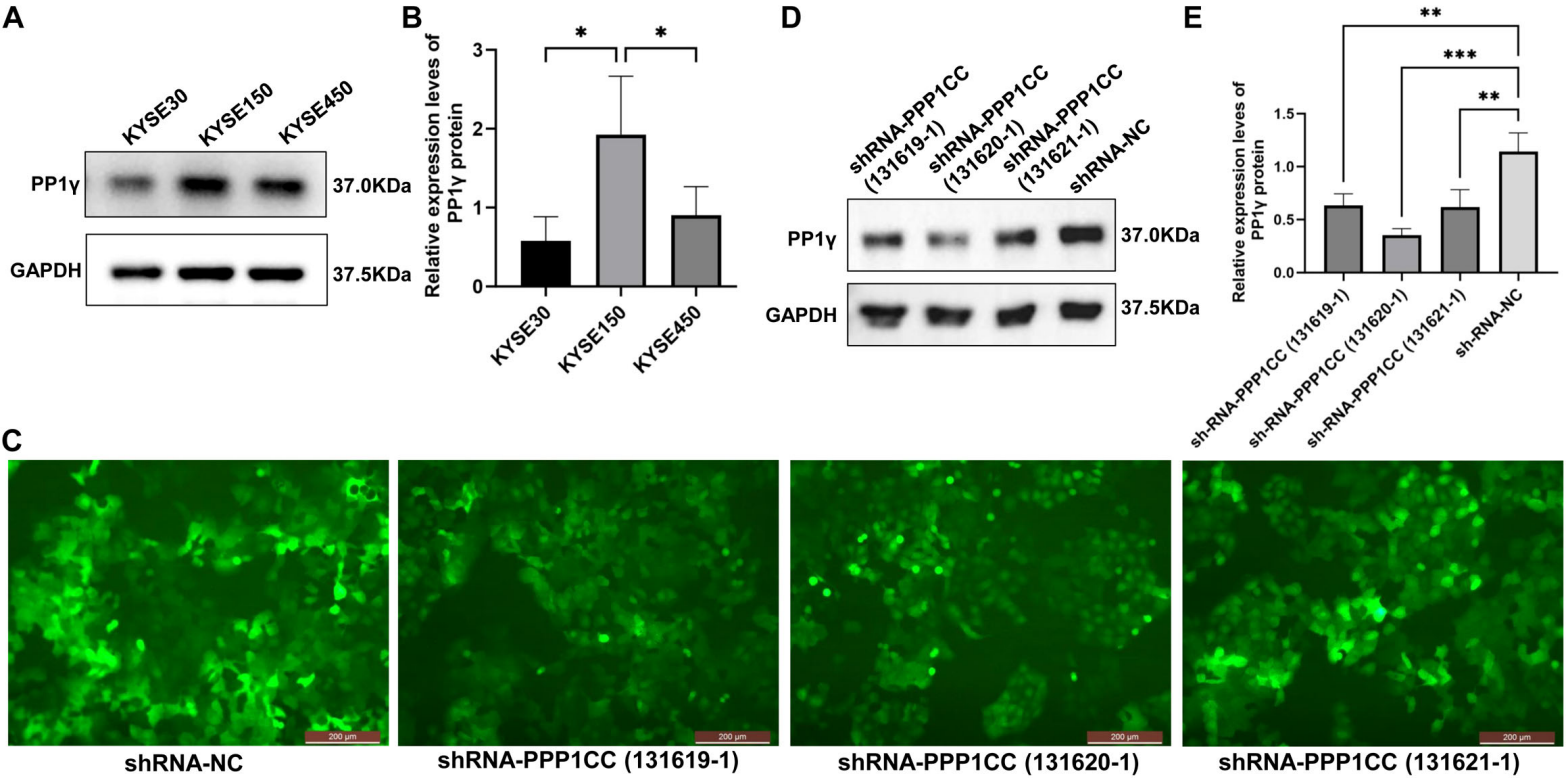
PPP1CC silencing using lentiviruses in cells. **(A, B)** Western blot detected the PP1γ protein in esophageal cancer cells. **(C)** The transfection efficiency was observed under an inverted fluorescence microscopy (Magnification × 200). **(D, E)** Western blot detected the PP1γ protein levels in cells transfected with shRNA-NC, shRNA-PPP1CC-131619-1, shRNA-PPP1CC-131620-1, and shRNA-PPP1CC-131621-1. *p < 0.05, **p<0.01; ***p<0.001.

We first detected the expression of PP1γ protein in KYSE30, KYSE150, and KYSE450 cells by using Western blot. The results showed that the PP1γ protein expression in KYSE150 cells was significantly higher than that in KYSE30 and KYSE450 cells (**Figures 6A, B**) (P < 0.05). Thus, the KYSE150 cell line was selected for the subsequent silencing of PPP1CC.

Next, the KYSE150 cells were transfected with shRNA-NC, shRNA-PPP1CC-131619-1, shRNA-PPP1CC-131620-1, and shRNA-PPP1CC-131621-1. As shown in **Figure 6C**, these three shRNA constructs produced strong GFP fluorescence signals, suggesting good transfection efficiency. Additionally, the silencing efficiency was evaluated by Western blot. The results revealed that the expression of PP1γ protein after shRNA-PPP1CC transfection was significantly decreased and the lowest level was observed in cells transfected with shRNA-PPP1CC-131620-1 (P < 0.05), suggesting the highest silencing efficiency with this shRNA (**Figures 6D, E**). Thus, shRNA-PPP1CC-131620–1 was used for subsequent experiments.

### 3.6 PPP1CC silencing inhibits cell proliferation, migration, and invasion of KYSE150 cells

**Figure 7:**
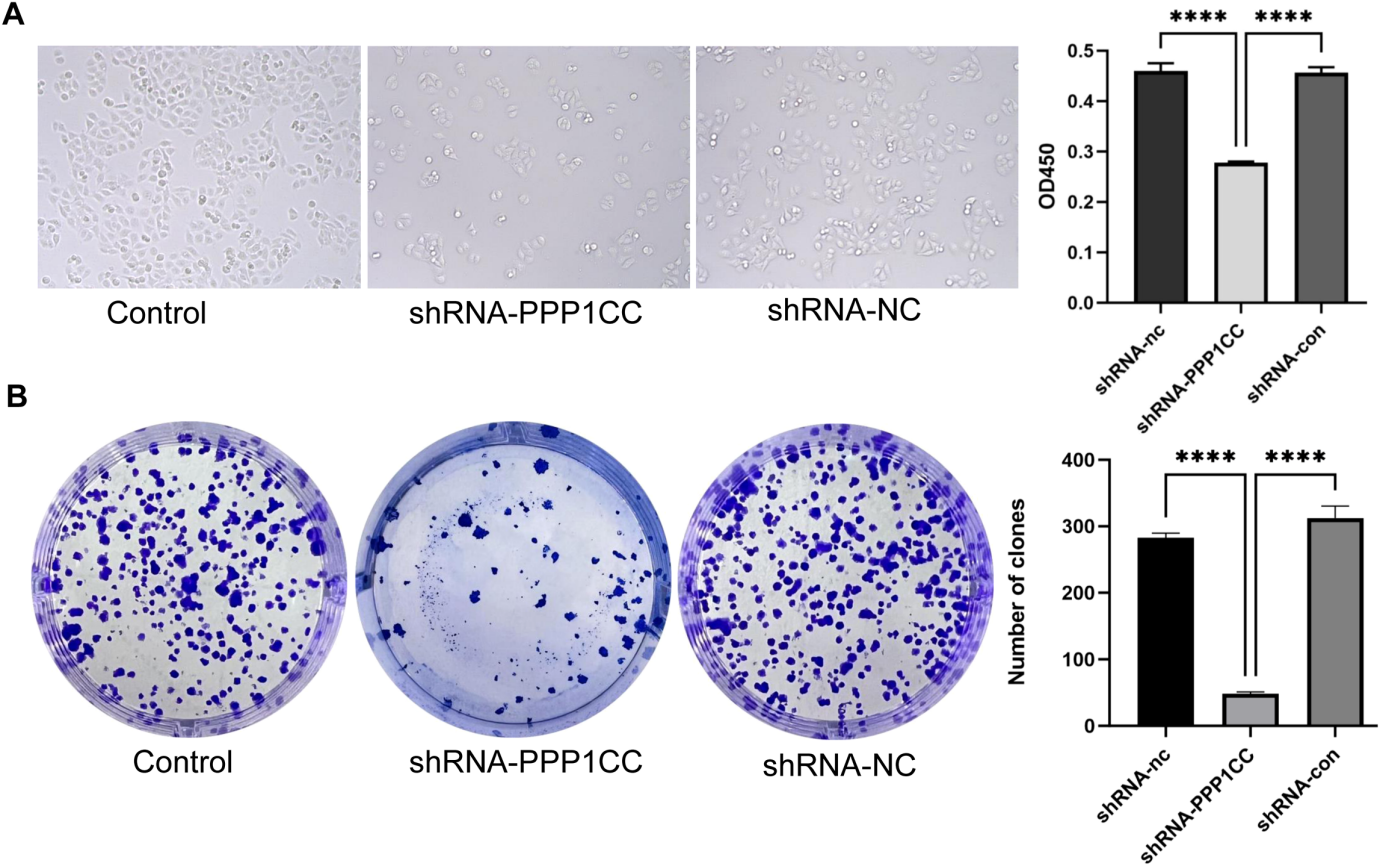
Effect of PPP1CC silencing on cell proliferation and colony formation. **(A)** The effect of PPP1CC silencing on the proliferation of KYSE150 cells was detected with the CCK-8 assay. The OD450 value was compared among groups. **(B)** The effect of PPP1CC silencing on the colony formation of KYSE150 cells. The number of colonies was counted and compared. This should be corrected into ****p<0.0001.

**Figure 8:**
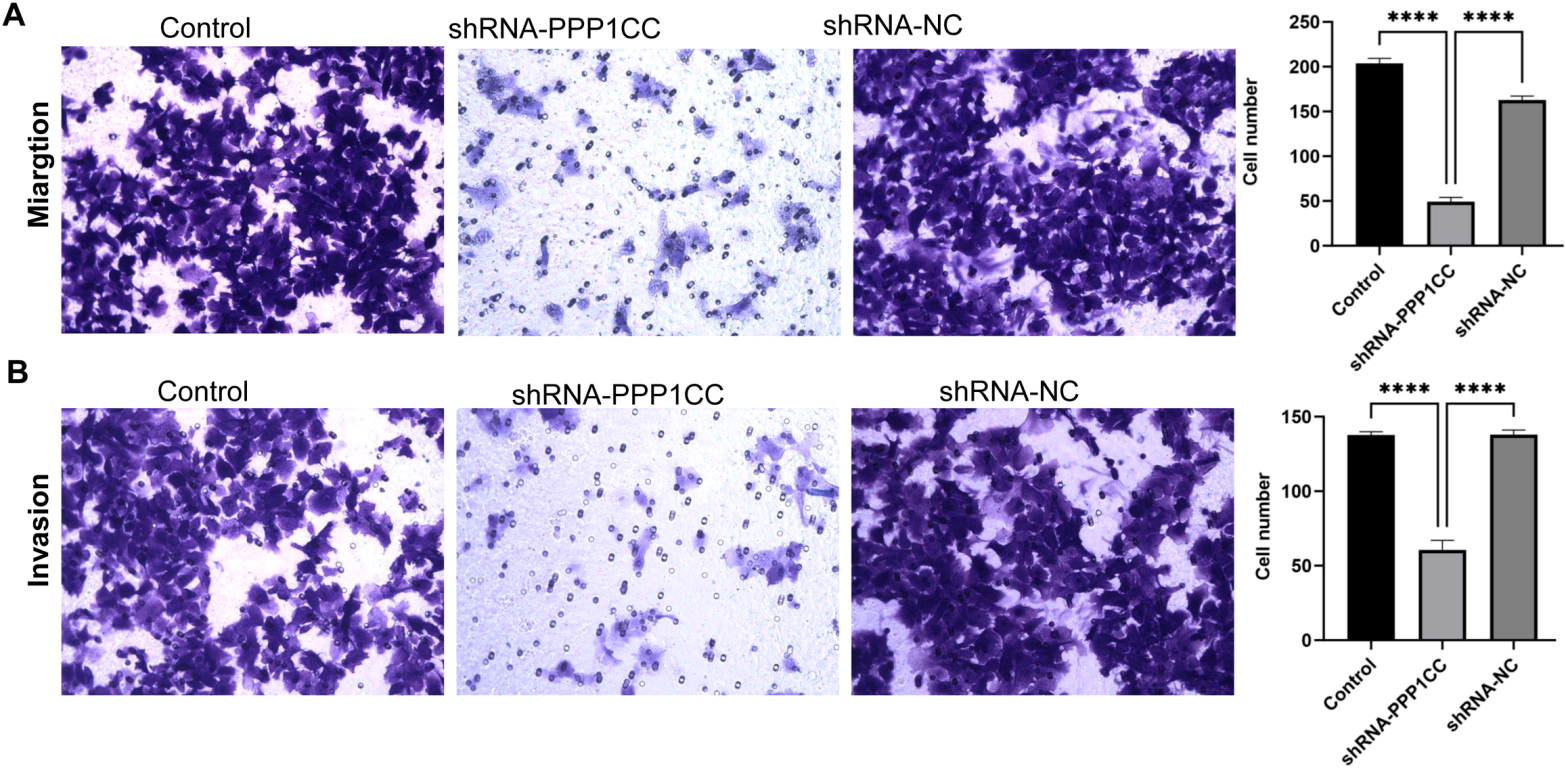
The effect of PPP1CC silencing on the migration and invasion of KYSE150 cells. Cell migration and invasion were assessed with the Transwell assay. **(A)** The results of cell migration. **(B)** The results of cell invasion. This should be corrected into ****p<0.0001.

To investigate the functional role of PP1γ in ESCC, we silenced PP1γ in the KYSE150 cell line using lentiviral transduction and then detected cell growth, migration, and invasion. CCK-8 assay revealed that the OD450 value in the shRNA-PPP1CC group was significantly lower than that in the control groups (P < 0.01) (**Figure 7A**). Similarly, colony formation assay demonstrated that the number of colonies in the shRNA-PPP1CC group was significantly decreased than that in the control groups (P < 0.01) (**Figure 7B**). Additionally, the Transwell assay further revealed that PPP1CC silencing resulted in a marked decrease in both the migration (**Figure 8A**) and invasion (**Figure 8B**) of ESCC cells (P < 0.01). These results showed that silencing PPP1CC suppressed the proliferation, migration, and invasion of ESCC cells, indicating its promotive effect of PP1γ on the aggressive behavior of ESCC.

### 3.7 Effects of PPP1CC silencing on YAP1, SOX2, and NANOG expression

**Figure 9:**
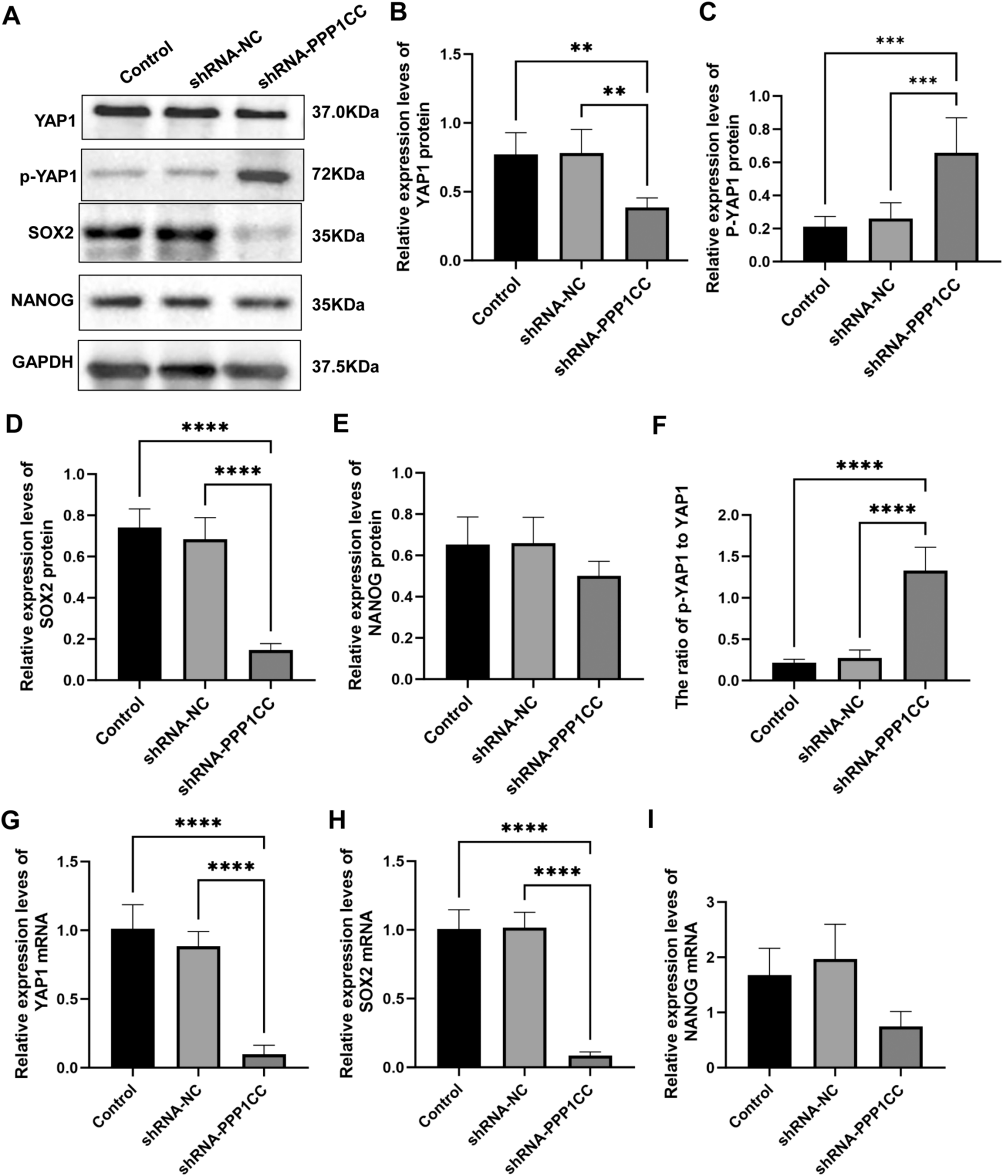
The effect of PPP1CC silencing on the expression of p-YAP1, YAP1, SOX2, and NANOG in KYSE150 cells. **(A-F)** The protein expressions of p-YAP1, YAP1, SOX2, and NANOG in KYSE150 cells were detected with Western blot. **(A)** Representative Western blot results. **(B)** Relative expression of YAP1. **(C)** Relative expression of p-YAP1. **(D)** Relative expression of SOX2. **(E)** Relative expression of NANOG. **(F)** The ratio of p-YAP1 to YAP1. **(G-I)** The mRNA expressions of YAP1, SOX2, and NANOG in KYSE150 cells were measured with qRT-PCR. **(G)** Relative expression of YAP1 mRNA. **(H)** Relative expression of SOX2 mRNA. **(I)** Relative expression of NANOG mRNA. ***p < 0.001; **p < 0.01, ****p<0.0001.

To detect the protein expression of YAP1, p-YAP1, SOX2, and NANOG in KYSE150 cells after silencing PPP1CC, Western blot analysis was performed. As shown in **Figures 9A, C**, PPP1CC silencing led to a significant increase in p-YAP1 levels, indicating inhibited YAP1 activity (P < 0.05). Moreover, the levels of YAP1 (**Figures 9A, B**) and SOX2 (**Figures 9A, D**) (P < 0.05). However, the changes in NANOG were not significant (**Figures 9A, E**) (P > 0.05). Additionally, the ratio of p-YAP1 to YAP1 was significantly increased after PPP1CC silencing (P < 0.05) (**Figure 9F**). Consistently, these results were further supported by qRT-PCR, which showed a significant decrease in *YAP1* (**Figure 9G**) and *SOX2* (**Figure 9H**) mRNA levels in the shRNA-PPP1CC group compared to controls (P < 0.05). Similarly, no significant change was observed in *NANOG* mRNA (**Figure 9I**) (P > 0.05). Collectively, these findings suggest that PP1γ may regulate YAP1 dephosphorylation and promote the expression of cancer stem cell markers SOX2 and NANOG, thereby enhancing the tumorigenic potential of ESCC cells.

## 4 Discussion

In this study, we investigated the role of PP1γ in the progression of ESCC and its modulation of YAP1 and cancer stem cell markers such as SOX2. Our findings revealed that PP1γ was significantly overexpressed in ESCC tissues compared to adjacent non-cancerous tissues, and this high expression correlated with advanced clinical features and poor patient prognosis. Silencing PPP1CC in ESCC cell lines resulted in decreased cell proliferation, migration, and invasion, alongside elevated levels of p-YAP1 and an increased ratio of p-YAP1 to YAP1, indicating reduced YAP1 activity. Furthermore, the downregulation of PP1γ led to a marked decrease in the expression of YAP1 and SOX2, while the effects on NANOG expression were not significant. Collectively, these findings suggest that PP1γ may play an important role in tumor invasiveness and metastasis of ESCC through the PP1γ/YAP1/SOX2 axis. Understanding the role of PP1γ provides insights into the molecular pathways involved in ESCC aggressiveness. Our findings may provide evidence for developing targeted therapies based on the PP1γ/YAP1/SOX2 axis for ESCC treatment.

PP1 plays a crucial role in the dephosphorylation of approximately 90% of eukaryotic proteins *in vivo* and is involved in regulating various functions associated with tumor progression, such as cell proliferation, migration, invasion, and metabolism. The catalytic subunit of PP1 is encoded by *PPP1CA*, *PPP1CB*, and *PPP1CC* (protein name PP1γ), and the roles of each subunit in tumor development are inconsistent (22, 23). In breast cancer, PP1 has been shown to interact with factors such as BRCA1 and DARPP-32, thereby playing a critical role in the tumor microenvironment and regulating processes such as cell migration (13). Inhibition of PP1 partially blocks the nuclear aggregation and activation of YAP1 in pancreatic cancer, suggesting that the PP1 cascade mediates the dephosphorylation and inactivation of YAP1 (24). Furthermore, MYPT1 enhances YAP1 dephosphorylation by interacting with PP1 in ovarian cancer, promoting nuclear translocation and subsequently increasing the expression of pro-survival genes while inhibiting apoptosis (15). PP1 can also dephosphorylate and inactivate LATS1/2, a kinase within the Hippo pathway, thereby reducing its inhibitory effect on YAP1 (25). This suggests that PP1 may indirectly regulate YAP1 through modulation of the Hippo pathway, highlighting the complexity of YAP1 regulation. Additionally, PP1 can directly bind to YAP1 through its catalytic subunits. For example, in colorectal cancer, PP1 can directly dephosphorylate Ser127 in YAP, facilitating YAP’s entry into the nucleus and contributing to tumor progression (26). However, the role and mechanism of PP1γ in ESCC have not been fully elucidated, particularly the mechanism of YAP1 dephosphorylation mediated by PP1γ. In this study, bioinformatics analysis revealed that PP1γ was overexpressed in ESCC and correlated significantly with age, lymph node metastasis, low tumor differentiation, and advanced clinical stages. Furthermore, a positive correlation was observed between PP1γ and the expression levels of YAP1 and SOX2, suggesting that high levels of PP1γ may facilitate the progression of ESCC through its interaction with these proteins. Immunohistochemical analysis confirmed the elevated expression of PP1γ in 107 ESCC paraffin tissue samples and its association with lymph node metastasis. Additionally, analysis of the TCGA database revealed that the OS and PFS were significantly shortened in ESCC patients with high PP1γ expression; thus, PP1γ was identified as an independent risk factor impacting the prognosis of ESCC.

YAP1, as a key effector of the Hippo signaling pathway, is frequently dysregulated in various cancers, including ESCC (27). It has been demonstrated that YAP1 acts as an oncogene in ESCC and is associated with poor prognosis, advanced stages, and metastasis (28). Consistently, the results of this study showed that the high expression of YAP1 was associated with male gender, late AJCC stage, and lymph node metastasis. Kaplan-Meier survival analysis found that the median OS and PFS of patients with high YAP1 expression were shorter than those of patients with low YAP1 expression. Additionally, there was protein interaction between PP1γ and YAP1.

To further explore the effects of PP1γ on the biological behavior of ESCC cells and its molecular mechanism, we silenced PP1γ in the KYSE150 ESCC cell line. The results showed that after PPP1CC silencing, the proliferation activity and the number of colonies were significantly reduced, suggesting that the low expression of PP1γ may inhibit the proliferation of ESCC cells. Furthermore, the migration and invasion ability of ESCC cells with low expression of PP1γ were also decreased. This is consistent with our findings in paraffin samples that PP1γ protein was highly expressed in ESCC tissues and associated with lymph node metastasis. The role of PP1γ in promoting the proliferation and invasion of various cancer cells has been established. For example, PP1γ is highly expressed in hepatocellular carcinoma tissues and is associated with tumor metastasis. Downregulation of PP1γ can lead to cell cycle arrest and reduced proliferation of hepatocellular carcinoma cells (29). Additionally, PP1γ has been found to bind to Ku70/Ku80 heterodimers in nasopharyngeal carcinoma, enhancing non-homologous end joining-mediated DNA repair and promoting tumor cell proliferation and migration (11). Our experimental results align with these findings, suggesting that PP1γ may play a critical role in the proliferation, migration, and invasion of ESCC, thereby influencing its progression. However, contrasting effects of PP1γ across various cancer types have also been reported. For example, in prostate cancer, the upregulation of PP1γ can lead to a reduction in AKT phosphorylation, thereby inhibiting cancer cell proliferation and migration by disrupting glycolytic metabolism and altering lipid metabolism (30). This contrasting role of PP1γ in different cancer types may be attributed to tissue-specific signaling networks or substrate selectivity.

Additionally, the high expression of cancer stem cell markers such as SOX2 and NANOG has been implicated in tumorigenicity and metastasis (31, 32). Moreover, it has been reported that YAP1 and SOX2 were highly expressed in ESCC, and their expressions were positively correlated (33). In this study, the protein interaction network indicated that PP1γ was closely correlated with YAP1, SOX2, and NANOG. Consistently, Spearman correlation analysis showed that PP1γ was positively correlated with the expression of YAP1, SOX2, and NANOG in ESCC samples. Our results also revealed that SOX2 and NANOG were highly expressed in ESCC, and their expressions were correlated with lymph node metastasis and the late pathological stage of the tumor. However, the expression of NANOG was not significantly changed at both the mRNA and protein levels in the context of PP1γ silencing *in vitro*. This raises intriguing questions about the regulatory mechanisms regulating NANOG expression in ESCC. As a core multifunctional factor, NANOG is subject to stringent regulation by various signaling pathways. While PP1γ-YAP1 may influence NANOG expression, silencing PP1γ alone may not sufficiently counterbalance other compensatory mechanisms. One potential pathway for this compensation involves the regulation of NANOG by PP1γ being offset by alterations in the activity of ubiquitin ligases (such as FBXW8) or deubiquitinases (such as USP21), thus maintaining the homeostatic level of NANOG protein (34, 35). Furthermore, SOX2, as a key interacting partner of NANOG, directly regulates its expression and mitigates the impact of PP1γ downregulation (36). Additionally, the epigenetic landscape of the NANOG promoter, particularly its hypomethylation status and the presence of active histone markers, can sustain transcriptional activity independently of YAP1 regulation (37, 38). Future studies aimed at exploring these regulatory pathways and potential post-translational modifications are essential for fully elucidating the implications of NANOG expression in ESCC.Reduced levels of the PP1 complex can lead to phosphorylation and activation of Akt, promoting epithelial-mesenchymal transition and enhancing the proliferation and metastasis of breast cancer stem cells (39). Our previous study has shown that PP1γ is highly expressed in glioma tissues, and this high expression is correlated with elevated levels of YAP1 and SOX2 (40). This suggests that PP1γ expression may influence the expression of YAP1 and SOX2, and is associated with the growth and malignant progression of glioma. Subsequently, we further investigated the mechanisms by which PP1γ affects ESCC and its correlation with YAP1 and SOX2. Western blot found that after PPP1CC silencing, the expression level of p-YAP1 protein was increased and the expression of YAP1, and SOX2 was decreased. Similarly, qRT-PCR results showed that the mRNA expression levels of YAP1 and SOX2 in PP1γ-silenced cells were significantly lower. Consistent with previous findings (26), the increase in p-YAP1 suggests that PP1γ may directly dephosphorylate YAP1, thereby promoting its nuclear translocation and transcriptional activity. Notably, the p-YAP1/YAP1 ratio significantly increased after silencing PPP1CC, which is consistent with the function of PP1γ as a protein phosphatase (41, 42). The PPP1CC silencing leads to a reduction of PP1γ, hindering the dephosphorylation of YAP1 and consequently resulting in the accumulation of p-YAP1 (2, 6, 43). Additionally, the decrease in total YAP1 levels may be attributed to changes in the stability of p-YAP1 or potential feedback regulation mechanisms. Interestingly, the reduction in PPP1CC expression not only resulted in decreased levels of p-YAP1 but also induced a significant decrease in YAP1 mRNA expression. One proposed mechanism for this decrease could involve the regulation of YAP1 transcription through downstream signaling pathways that interact with PP1CC. The dephosphorylation of specific transcriptional co-activators by protein phosphatases may enhance their ability to activate or repress target genes, including YAP (44, 45). For instance, PP1 has been shown to play a crucial role in regulating the stability of transcription factors and subsequently their mRNA levels (46, 47). This mechanism is further supported by our findings from the TCGA-esophageal cancer cohort, which revealed a positive correlation between PPP1CC and YAP1 mRNA expression, suggesting a potential link between PP1CC expression and YAP1 transcription. Collectively, PP1γ downregulation increased the phosphorylation level of YAP1, affecting the expression of YAP1 and SOX2.

This study has some limitations. First, our results were primarily derived from a single ESCC cell line, which may limit the generalizability of these results. Future studies should include a broader range of ESCC cell lines, *in vivo* xenograft models, or patient-derived samples to confirm our findings. Second, although YAP1 and SOX2 were identified as downstream targets of PP1γ, other potential pathways or interacting proteins that may also contribute to PP1γ-mediated effects have not been explored. Further studies are warranted. Third, only one shRNA was used for the silencing experiments, which may limit the robustness of our findings due to the potential for off-target effects. Future studies would benefit from the use of multiple shRNA constructs targeting PP1γ to strengthen the validity of the observed effects and minimize the risk of confounding results.

In conclusion, our study identifies PP1γ as a key regulator of YAP1 dephosphorylation and stem cell marker expression in ESCC, promoting tumor growth, invasion, and metastasis. The PP1γ/YAP1/SOX2 axis represents a potential therapeutic target for improving outcomes in ESCC patients, particularly those with advanced disease. Future research should focus on validating these findings in other ESCC models, including patient-derived xenografts, to better understand the clinical implications of targeting PP1γ. Additionally, exploring the broader network of PP1γ-regulated pathways and interactions with other signaling molecules will provide a more comprehensive understanding of its role in ESCC. Investigating the efficacy of PP1γ inhibitors or YAP1-targeting therapies in preclinical models will be crucial for determining the potential of this pathway as a therapeutic target.

## Data Availability

The raw data supporting the conclusions of this article will be made available by the authors, without undue reservation.
